# Diet type influences the gut microbiome and nutrient assimilation of Genetically Improved Farmed Tilapia (*Oreochromis niloticus*)

**DOI:** 10.1371/journal.pone.0237775

**Published:** 2020-08-19

**Authors:** Lara Parata, Debashish Mazumder, Jesmond Sammut, Suhelen Egan

**Affiliations:** 1 Centre for Marine Science and Innovation, School of Biological Earth and Environmental Science, University of New South Wales, Sydney, NSW, Australia; 2 Australian Nuclear Science and Technology Organisation, Kirrawee DC, NSW, Australia; Universitetet i Nordland, NORWAY

## Abstract

Nile tilapia, *Oreochromis niloticus* is the third most commonly farmed finfish species in the world, accounting for nearly 5% of global aquaculture production. In the past few decades much of the success of this species has been attributed to the development and distribution of Genetically Improved Farmed Tilapia (GIFT). Despite the increasing availability of GIFT, the productivity of small-scale farming remains highly variable, particularly in developing nations. Commercial fish-feed pellets can increase fish farm productivity; however, many small-scale farmers rely on other means of feeding fish due to the high cost and limited availability of commercial fish feed pellets. Therefore, understanding how locally-sourced feeds affect the production of GIFT is an important step towards improving feeding practices, particularly for farmers with low financial capital. This study used stable isotope analysis (SIA) and 16S rRNA gene sequencing to compare the effects of a locally-sourced vegetable-based diet and commercial pellet-based diets on the relative condition, nutrient assimilation patterns and gastrointestinal microbiota of GIFT. GIFT fed a locally-sourced diet were smaller, and in a significantly poorer condition than those fed with commercial fish feeds. SIA showed no differences in dietary carbon between the two diets; however, δ^13^C, poor fish condition and the abundance of specific bacterial taxa (of such as Fusobacteria) were correlated. SIA revealed that GIFT fed locally-sourced diets that predominantly consisted of vegetables were significantly enriched in δ^15^N despite a perceived lack of dietary protein. This enrichment suggests that GIFT fed a locally-sourced diet may be supplementing their diet via cannibalism, a behaviour representative of poor farming practice. Overall this study highlights the need to increase the availability of suitable GIFT feeds in developing nations. The development a low-cost feed alternative could improve the success of small-scale GIFT farmers in PNG, increasing both food and income security within the region.

## Introduction

Tilapia are amongst the most important aquaculture species of the 21^st^ century accounting for 10% of the world’s finfish production [1]. Nile tilapia (*Oreochromis niloticus*) is the most commonly farmed tilapia species accounting for 83% of tilapia production through both commercial and small-scale aquaculture [2]. WorldFish selectively bred *O*. *niloticus* to develop the Genetically Improved Farmed Tilapia (GIFT) [3–5]. The global impact and success of GIFT has been particularly evident in developing nations where it has helped to improve food and income security [4, 6].

Inland aquaculture in Papua New Guinea (PNG) is mostly small-scale subsistence farming with growth limited by infrastructure, the high cost and limited availability of commercial feed, the poor economic status of people and a lack of fish husbandry skills in the farming communities [7–9]. The farming of GIFT has been a greater success in PNG compared to other fish species, such as trout, because it is a lower maintenance species and easy to breed [10]. Nevertheless, the ruggedness of PNG’s interior, where GIFT is mostly farmed, makes fish farming a challenge. As for many farmed species, commercial fish feed pellets are widely considered the best option to increase GIFT farm productivity; however, only 10% of the small-scale fish farmers in PNG use commercial fish feed [10]. Whilst the nutritional needs of farmed tilapia can be met through a variety of natural food sources [11], a poor diet can negatively impact their growth and overall health [12].

The productivity of small-scale fish farms in PNG and other developing countries could be increased by better understanding the dietary preferences and nutrient assimilation patterns of the fish. Previous studies have relied on gut content analysis to classify the diet of fish [13]; however, gut content analysis does not determine if the consumed food contributes to growth. In recent years, stable isotope analysis (SIA) has been used [14] to determine the source of assimilated nutrients that contribute to growth [15, 16]. SIA depicts average nutrient assimilation patterns based on the estimated turnover rate of the stable isotopes sourced from food, allowing for accurate inferences to be made about a consumer’s diet [17]. Furthermore, analysis of the stable carbon and nitrogen isotopes can provide information regarding the trophic ecology of the fish [18–20] providing unique insights into the environmental influences and food web structure.

Knowledge of the composition and microbial diversity within the gastrointestinal tract is vital because of the influence these symbionts have on the host’s growth and survival [21–23]. For aquatic species, including finfish, farming practices can heavily influence the gastrointestinal microbiota, impacting digestion and the assimilation of essential nutrients [24–26]. Recent studies on tilapia microbiomes have generated data on the effects of dietary supplementation [27, 28] and rearing conditions [29, 30]; however, little is known on the effects of locally-sourced feeds. This study aimed to determine how different feeding practices affect the condition, nutrient assimilation patterns and the gastrointestinal microbiome of GIFT reared in earthen ponds. We aimed to use stable isotope analysis of carbon and nitrogen to identify differences in diets and therefore the trophic status of GIFT. Additionally, we aimed to associate specific microbial assemblages with different conditions, assimilation patterns and feeding practices to underpin the development of more effective farming practices for small-scale fish farmers.

## Methods

### Ethics statement

All experimental procedures involving live fish were approved by the University of New South Wales Animal Care & Ethics Committee (UNSW ACEC) under permit number 18/26B specifically for this study.

### Study site

*Oreochromis niloticus* (GIFT Strain) individuals were collected from six different fish farms within the Aiyura valley (-6.3381° S, 145.9042° E), located in the Eastern Highlands of Papua New Guinea. The stocked GIFT were from the same family line acquired from the Highlands Aquaculture Development Centre (HAQDEC) breeding program. The sampled farms all stock GIFT in earthen ponds, and represent one of two different feeding practices; a locally-sourced raw vegetable-based diet (mostly sweet potato, banana leaves and garden waste) (n = 3) (hereafter referred to as a ‘vegetable’ diet) and a mixed diet consisting of both the occasional supplementation of raw vegetables (mostly sweet potato, banana leaves and garden waste) and regular commercial feed pellets (n = 3) (hereafter referred to as a ‘pellet’ diet). The commercial fish feed pellets were all from a single imported source from Vietnam and included 30% crude protein, 5% crude fat, 16% ash, 6% crude fibre and 11% moisture with raw ingredients including fishmeal, wheat flour, soybean meal, fish oil, rice bran and vitamins and minerals. Parameters such as feeding frequency and pond size were recorded on site during sample collection. The age of the GIFT and stocking density of the sampled ponds was estimated as there were no records kept of fish being removed from the ponds (for consumption or the sale at local markets) or restocking events. Water quality measurements of temperature (n = 3), pH (n = 3) and dissolved oxygen (DO) (n = 3) were recorded at each farm using a TPS WP91 water quality meters with an Ionode J44 intermediate junction pH probe and YSI DO probe. Probes were calibrated daily according to the manufacturer’s guidelines and standards.

### Sampling

Individual fish (n = 10) of similar size were collected from each farm using handheld nets and immediately euthanised in an AQUI-S solution (UNSW Animal Care & Ethics Committee Permit: ACEC number 18/26B). All fish were devoid of any gross or clinical signs of disease. Standard measurements of length and weight were recorded prior to dissection. Dissections were undertaken at HAQDEC within 2 hours of collection. White dorsal tissue samples were aseptically removed, scaled and skinned before being rinsed with distilled water and stored at -20°C. In addition, the gastrointestinal tract was aseptically removed with a combined hindgut content and hindgut wall sample collected and frozen (initially at -20°C) and stored at -80°C prior to microbial analysis.

### Stable isotope analysis

White dorsal tissue samples were prepared and analysed as per the methods previously described by Kinney, Hussey [31] and Gopi, Mazumder [32]. Briefly, dorsal tissue samples were washed with distilled water, oven-dried, ground to fine powder and loaded into tin capsules for analysis. Powdered samples were analysed in the continuous flow isotope ratio mass spectrometer (CF‐IRMS), model Delta V Plus (Thermo Scientific Corporation, USA), at the Australian Nuclear Science and Technology Organisation (ANSTO) for stable carbon and nitrogen isotopes. The data were reported relative to IAEA (International Atomic Energy Agency) secondary standards, and all results were certified relative to air for nitrogen, and Vienna-PeeDee Belemnite (VPDB) for carbon. The results were accurate to 1% for both C% and N% and ±0.3 parts per thousand (‰) for δ^13^C and δ^15^N. The C: N molar ratio relates to the lipid content of a sample and affects δ^13^C values. When the C:N ratio was greater than 3.5, the result was mathematically corrected to account for the lipid content using the formula specified by Post, Layman [33] as follows:

$$\delta^{13}C_{corrected}=\delta^{13}C_{untreated}-3.32+0.99\times C:N$$

### 16S rRNA gene amplicon sequencing and analysis

Bacterial DNA was extracted from the hindgut samples (8 mg) using the DNeasy® Blood &Tissue Kit (QIAGEN, USA) following the manufacturer’s protocol. The diversity and structure of the microbial community associated with each sample was assessed by amplicon sequencing of the V3-V4 variable region of the 16S rRNA gene, using the barcoded universal bacterial primers: 341F (CCT ACG GGN GGC WGC AG) and 785R (GAC TAC HVG GGT ATC TAA TCC) [34]. PCR amplification was performed using the following conditions; 94°C for 2 minutes, followed by 40 cycles of 94°C for 30 seconds, 50°C for 30 seconds and 72°C for 30 seconds with a final extension of 72°C for 7 minutes. Barcoded samples (N = 60) were sequenced on a MiSeq Illumina (2 x 300 bp) at the Ramaciotti Centre for Genomics (UNSW Sydney, Australia) generating paired-end reads of approximately 440 base-pairs (bp).

Firstly, sequencing reads of low quality (quality score of less than 15 [35]) were removed using Trimmomatic version 0.38 [35]. Subsequent processing of the reads was conducted using Usearch version 11.0.667 [36]. Paired-end reads were merged, and quality filtered to remove reads with more than five errors and less than 300bp. The resulting sequences were then trimmed to remove primer sequences before being clustered in zero-radius operational taxonomic units (zOTU). A *de novo* chimera removal was included in the clustering step with remaining chimeras removed using the UCHIME algorithm with reference to the SILVA reference database (SILVA SSURef 132 NR) [37]. zOTU sequences were then taxonomically classified using UCHIME by BLAST alignment against the SILVA database. Samples with low zOTU counts (< 30, 000) were removed. The zOTU counts of each sample were rarefied through random sampling to a value of 34, 313 (lowest count) to account for uneven sequencing depth across samples using the R Package ‘vegan’ [38]. Rare zOTUs with counts across all samples of <25 were removed after rarefaction. After removal of samples and zOTUs, as described above, the resulting count table consisting of 1,830 zOTUs and their counts in each sample (N = 52, S1 Table) was used for all subsequent analysis.

### Statistical analysis

All data analyses were conducted in R version 3.4.3 [39]. Relative condition factor (K_n_) was used to assess the condition of the sampled fish and was calculated as described in Jisr, Younes [40] and Le Cren [41]. Two fish were identified as significant outliers (>3 SD and > 95% CI) and therefore removed from the condition and subsequent correlation analysis. Analysis of variance (ANOVA) was used to test for significant differences in relative condition, water quality, weight, total length, standard length and isotopic values (δ^13^C and δ^15^N) of GIFT between the two feeding practices. Differences isotopic values were visualised using the package ggplot2 version 2.2.1 [42].

Alpha diversity (Shannon Weaver Index) was calculated in the ‘vegan’ package version 2.5–4 with statistical differences analysed using a one-way ANOVA [38]. Differences in community structure were investigated by permutational multivariate analysis of variance (PERMANOVA) based on Bray-Curtis distance matrices using the ‘vegdist’ and ‘adonis’ functions in the ‘vegan’ package. Additionally, differences in community structure were visualized using the metaMDS function. Differences in dispersions (variances) between treatments were determined by Levene's test for homogeneity of variances using ‘betadisper’.

To identify specific zOTUs that significantly correlated with relative fish condition and nutrient assimilation patterns, we assessed those with a relative abundance of over 1% that were present in at least 90% of the samples. Correlation coefficients were calculated using Pearson’s correlation with a significance threshold of P < 0.05.

To identify specific zOTUs associated with each of the farming practices, multipattern analyses were conducted using the ‘multipatt’ function within the ‘indicspecies’ package version 1.7.6 [43]. Indicator species values were calculated using the ‘indval’ function. Correlation indices (phi coefficients) were calculated using the r.g function [44]. This calculation used presence-absence data and point-biserial correlation coefficients corrected for unequal sample sizes [44]. The zOTUs that were identified as significant indicators or associated with significant correlation indices were visualised using ‘pheatmap’ version 1.0.10 [45]. The heatmap results were visualised and z-score transformed to represent the number of standard deviations a zOTU abundance was from the overall mean abundance of that zOTU.

## Results

### The condition of Genetically Improved Farmed Tilapia

GIFT fed a diet incorporating commercial pellets were in a significantly better condition than those only fed vegetables (Table 1). Pellet-fed GIFT were also significantly heavier and larger than their vegetable-fed counterparts with significant differences observed for both total and standard length (Table 1). Dissolved oxygen concentrations were significantly lower in farms that used only a vegetable-based diet when compared to farms using commercial feeds (Table 1). There was no significant difference in temperature or pH between the two farming practices nor was there a significant correlation with fish condition (Table 1).

**Table 1:** ANOVA results showing a comparison of growth statistics and water quality parameters for *O*. *niloticus* farmed under different diets.

	Average (±SD)		*F statistic*	*Significance*
	Vegetable-based	Pellet-based		
Standard Length (cm)	10.95 (2.31)	16.67 (3.59)	49.1	<0.01
Total Length (cm)	13.59 (2.76)	19.70 (3.21)	57.17	<0.01
Weight (g)	51.80 (32.81)	151.34 (72.85)	43.35	<0.01
Relative condition (kn)	0.98 (0.11)	1.03 (0.18)	5.482	0.02
Dissolved Oxygen	4.65 (3.57)	10.48 (2.27)	10.9	0.03
Temperature (ºC)	24.02 (3.26)	22.47 (1.74)	1.594	0.225
pH	7.23 (0.69)	7.82 (0.65)	3.454	0.0816
Feed frequency (n / week)	3.67 (2.89)	7 (0)	4	0.116
Fish Age* (years)	2.17 (1.04)	2 (1.80)	0.019	0.896
Pond surface area (m^2^)	46 (15.1)	354 (387.49)	1.893	0.241
Stocking density ** (fish/m^2^)	22.46 (12.8)	22.8 (24.26)	0.001	0.983

‘Pellet’ is representative of GIFT fed commercial fish feed pellet based diet, and ‘Vegetable’ is representative of GIFT fed a locally-sourced vegetable-based diet.

*Fish age is an estimate provided by the farmers based on stocking information.

** Stocking density was estimated using information regarding pond size and initial stocking numbers from farmers.

### Stable carbon and nitrogen isotopes

**Fig 1:**
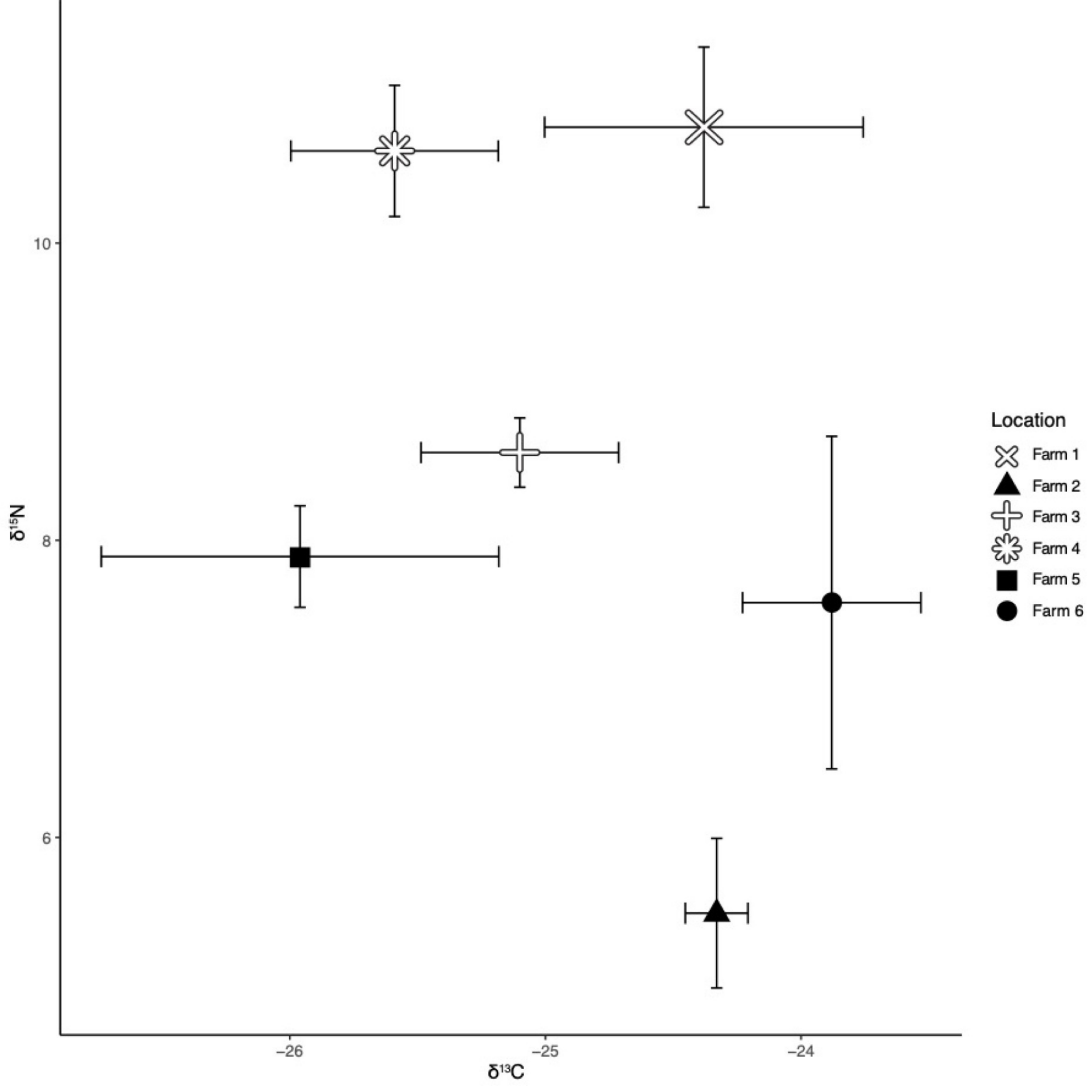
Stable isotope biplot of *O*. *niloticus*. Stable isotope biplot (mean and standard deviation of δ^13^C and δ^15^N values) of *O*. *niloticus* for each of the sampled farms. Pellet-fed fish are represented by solid, black shapes with vegetable-fed fish represented by outlined symbols.

Stable carbon isotope values did not vary significantly between vegetable and pellet-fed GIFT (Table 2), with the observed δ^13^C values for both feeding practices showing some overlap (Fig 1) and ranging between -27.1‰ and -23.2‰. The SIA data did show that the stable nitrogen isotope differed significantly (Table 2) between the treatments or diet types, and that the vegetable-fed GIFT were enriched in δ^15^N (Fig 1). The δ^15^N values ranged between 4.8‰ and 9.9‰ for pellet-fed GIFT and 8.1‰ and 11.6‰ for vegetable-fed GIFT.

**Table 2:** The ANOVA results for stable isotope values of *O*. *niloticus*.

	Average (±SD)		*F statistic*	*Significance*
	Vegetable-based	Pellet-based		
δ^13^C	-25.02 (0.81)	-24.74 (0.95)	1.5618	0.217
δ^15^N	10.00 (1.33)	7.04 (2.09)	91.043	<0.01

### Gut microbiome diversity associated with Genetically Improved Farmed Tilapia

High throughput amplicon sequencing of the 16S rRNA gene identified 1, 830 zOTUs among 52 samples. Rarefaction curves of the samples (subsampled to 34, 313 counts) indicated that all were sequenced nearly to saturation and hence their bacterial communities were well covered (S1 Fig).

**Fig 2:**
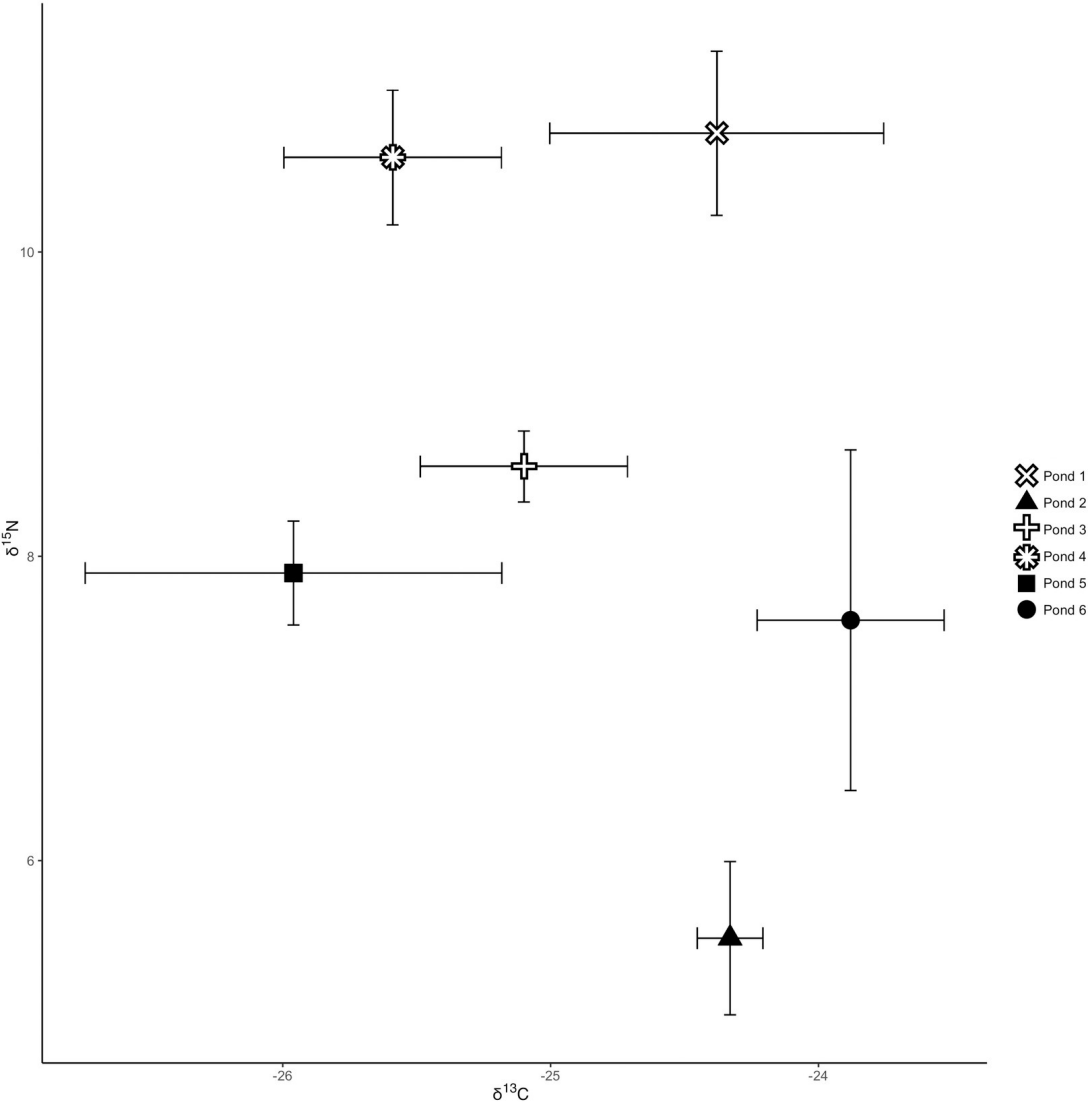
Bray-Curtis dissimilarities of the GIFT microbiome. Non-Metric multidimensional scaling (nMDS) plot based on Bray-Curtis dissimilarity of the bacterial communities of GIFT fed different diets (*P* = 0.001). Pellet-fed fish are represented by solid, black shapes with vegetable-fed fish represented by red symbols. Stress = 0.1703505.

Alpha diversity, as measured by the Shannon-Weaver Index, did not vary significantly between the two feeding practices (*P* = 0.683, S2 Fig). Ordination of the Bray-Curtis dissimilarities (Fig 2) showed a distinct separation between the bacterial assemblages of pellet and vegetable-fed GIFT. The bacteria associated with pellet and vegetable-fed GIFT were significantly different in overall species composition (*F* = 7.0596, *P* = 0.001) and dispersion (*F =* 4.0541, *P =* 0.04). There was more significantly variability in the clustering pattern of microbial communities associated with the vegetable diets than the more tightly clustered pellet associated communities (Fig 2, *F =* 4.0541, *P =* 0.04). Differences in species composition within each diet group (i.e vegetable and pellet) were also seen (Fig 2), with the dispersion of microbial communities associated with pellet-fed fish differing significantly between farms (*F =* 7.4513, P = 0.003).

Pearsons correlation revealed that three zOTUs (13, 2 and 9) were significantly correlated with fish condition and nutrient assimilation (Table 3). zOTUs 13 and 9 were both identified to the genus level as *Cetobacterium* sp. (Phylum: Fusobacteria) and were negatively correlated with fish condition and dietary carbon. Similarly, significant negative correlations were also identified for zOTU 2 (genus: *Fusobacterium* sp., Phylum: Fusobacteria).

**Table 3:** Pearson correlation coefficients between the relative condition (kn), dietary carbon (δ^13^C) and the relative abundance of bacterial taxa associated with *O*. *niloticus*.

	Relative condition (kn)		δ^13^C	
	Correlation coefficient	Significance	Correlation coefficient	Significance
Zotu2 (g Fusobacterium)	-0.49	<0.01	-0.41	<0.01
Zotu9 (g Cetobacterium)	-0.39	<0.01	-0.33	0.02
Zotu13 (g Cetobacterium)	-0.43	<0.01	-0.37	<0.01

**Fig 3:**
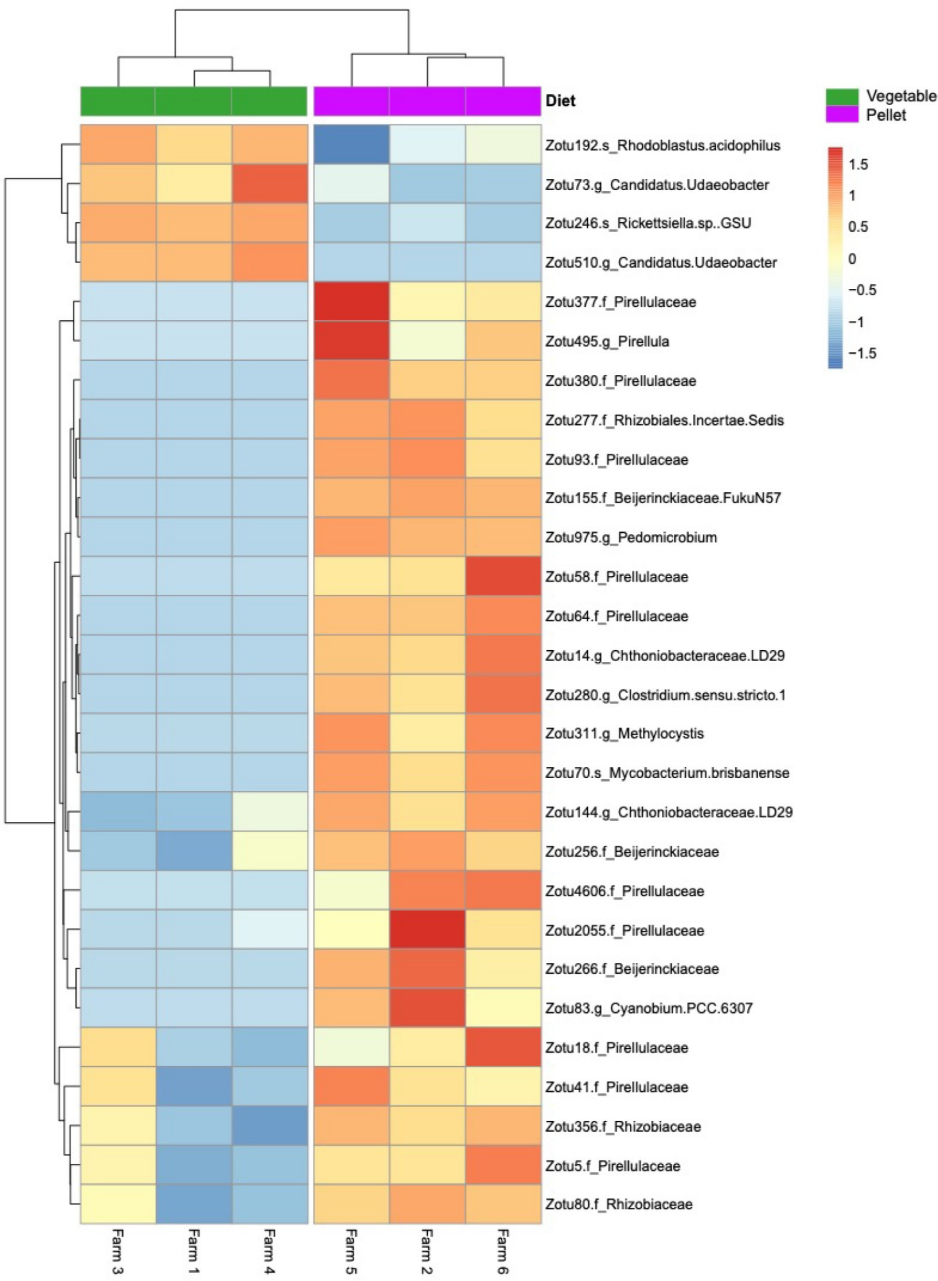
Bacterial taxa that are significant indicators of diet. Differentially abundant log-transformed zOTUs (identified to the lowest taxonomic level possible) (P-Adj <0.05) that represent the taxa that were found to be significantly indicative or associated with either the pellet, or vegetable-fed GIFT. zOTU abundances have been z-score transformed and thus show the number of standard deviations a zOTUs abundance is from the mean abundance of that zOTU.

Indicator species analysis revealed that 24 zOTUs were significantly associated with pellet-based diets (*P* <0.05) based either on presence and absence data, species abundances or both (Fig 3). Pirellulaceae was the most commonly identified family within this subset accounting for 45% of the zOTUs (Fig 3). Within this family, zOTU 5, 64 and 93 were found to be good indicators of pellet diets. In contrast, 4 zOTUs showed a significant association with tilapia fed vegetable diets (Fig 3). These bacteria were identified as *Rhodoblastus acidophilus* (zOTU 192) and *Rickettsiella sp*. *GSU* (zOTU 246) and *Candidatus Udaeobacter* (zOTU 73 and 510).

## Discussion

The isotopic values of a consumer are related to its diet; therefore, stable isotope analysis can be used to accurately identify a consumer’s dietary profile and trophic status [17, 18, 46]. The δ^13^C values for both the vegetable only and pellet-fed GIFT indicates some similarities in dietary carbon sources [47, 48]. Assessing dietary carbon is important, as it encompasses essential nutrients such as carbohydrates and lipids [49]. These nutrients are vital for fish health as they play an important role in growth and metabolism [50, 51]. In fish, fluctuations in dietary carbon are often reflected by their gastrointestinal microbiota [52]. In our study, an overlap in dietary carbon may be due to the occasional provision of vegetables to pellet-fed fish; however, a clear separation in δ^15^N suggests distinct dietary nitrogen sources. In aquatic systems, including aquaculture ponds, significantly enriched δ^15^N values can be indicative of anthropogenic nitrogen input [53] such as fertilisers [54]. Whilst the remoteness of our study site and absence of intensive agricultural farming reduces the potential for such anthropogenic inputs, some farmers do use chicken manure to fertilise crops. Small-scale piggeries and vegetable cropping also occur in the catchment of the farms, and urban activities in the nearby town of Kainantu, may also be sources of nutrients; however, their contribution to the nutrient budgets of the farms is likely to be negligible. Despite the use of chicken manure, a previous study has shown that this type of organic fertiliser does not influence the growth and δ^15^N of GIFT [11]. This indicates that the enrichment of δ^15^N in vegetable-fed GIFT in our study is not a result of fertiliser, rather, it likely represents a response to dietary preferences [55].

High δ^15^N values are regularly associated with an increase in the consumption of animal-based protein [49, 56]; however, this is not always indicative of an optimal diet [57]. In our study, vegetable-fed GIFT were significantly enriched in δ^15^N despite a perceived lack in dietary protein in the introduced food sources. An optimal diet for GIFT between 20-200g would consist of 34% protein [58]; however, a vegetable-based diet consisting of mostly sweet potato would likely provide the fish 1-5g of protein per 100g consumed [59, 60]. One explanation for our results is that the vegetable-fed GIFT are not receiving enough dietary nitrogen to meet their metabolic needs, and subsequently they are forced to utilise nitrogen reserves in the body, increasing the δ^15^N in their tissues [61]. Alternatively, provision of an insufficient diet may have resulted in the vegetable-fed GIFT supplementing their diet via cannibalism [62].

Within pond cannibalism can negatively affect a farm’s ability to produce table-sized fish and can therefore negatively affect productivity and profitability. Whilst fish cannibalise for many reasons, stress, limited food availability and low dissolved oxygen are considered major drivers [63–65]. Previous studies on tilapia have reported filial (both egg and fry) cannibalism, with stunted individuals, or progeny from the initial stocked fish, more likely to become prey [63, 65]. In our study, vegetable-fed GIFT averaged 6cm smaller and 100g lighter than their pellet-fed counterparts, further supporting the possibility of within pond cannibalism. It should be noted that these differences may also be attributed to age, as fish reproduction in farms is not traditionally controlled and was therefore not considered. While no direct evidence of cannibalism was observed, the organic material in the hindgut of the gastrointestinal tract of fishes is usually in an advanced stage of digestion, thus making it difficult to visually identify what was consumed [66].

The ability of a fish to effectively absorb nutrients and digest foods depends on its gastrointestinal microbiota [24–26]. The gastrointestinal microbiota can impact a fish’s weight and overall health [21, 67, 68]. Overall, we identified significant differences in the gastrointestinal microbiota of GIFT in response to feeding practice. This result is in accordance with previous studies that have reported changes in the microbiome of fishes in response to changes in diet, and dietary supplementation [27, 28]. In our study, a larger number of zOTUs were identified as significantly associated with commercial pellet-fed GIFT than those fed vegetables (Fig 3), likely reflecting diet stability as has been seen for humans and ants [69, 70]. Farmers using a commercial pellet-based diet for their GIFT consistently source imported tilapia feeds directly from the National Fisheries Authority (NFA). In contrast, the vegetable-only diet is inconsistent and largely determined by harvesting season and the availability of vegetable garden waste.

Pirellulaceae was the most commonly identified family within the taxa found to be significantly indicative of pellet-fed GIFT accounting for 45% of the zOTUs (Fig 3). Pirellulaceae (Phylum: Planctomycetes) are aquatic bacteria found in both marine and freshwater environments. Pirellulaceae have been previously identified in association with soils [71] and African cichlids [72]. Whilst little is known about the exact role of this family, a previous study has suggested that heterotrophic Planctomycetes play an important role in the fermentation of carbohydrates [73]. Carbohydrates account for a large portion of commercial fish feeds due to their low cost and binding properties [74]. The high abundance of this taxon within the gastrointestinal tract of GIFT suggests that they may be functioning as mediators in the breakdown and digestion of consumed carbohydrates.

*Candidatus Udaeobacter* copiosus sp. (phylum: Verrucomicrobia) was the most common taxon found to be significantly associated with GIFT fed a vegetable-only diet (Fig 3). *Ca*. *U*. copiosus is a relatively newly described bacterial genus that appears well adapted to the soil environment [75]. Thus, the presence of *Ca*. *U*. copiosus within the GIFT gastrointestinal tract is most likely a reflection of the tendency of these fish to consume detrital material [76], with this genus merely a transient taxon within the GIFT microbiota.

The presence of nine bacterial taxa across most (90%) of the fish in our study is of interest as it implies that these taxa may have been acquired from the hatchery prior to distribution. This finding is significant because the microbial composition of larval and juvenile fish has a significant influence of the microbiome of adults [77]. Three of these ‘hatchery associated’ taxa (zOTU 13, 2 and 9, Phylum: Fusobacteria) were negatively correlated with relative fish condition and δ^13^C (Table 3). These results suggest that depleting the availability of δ^13^C could decrease the abundance of Fusobacteria, and subsequently improve the relative fish condition of GIFT. Modification of δ^13^C within the fish tissue could be achieved by simply altering the types of vegetable-based foods used or promoting a farming practice that incorporates commercial fish-feed pellets as a ‘supplement’. Our study has shown that farming practices incorporating commercial-feed pellets increase relative fish condition of GIFT (Table 1). The promotion of a commercial-feed supplemented farming strategy would need to be incorporated not only by the fish farms, but also the hatcheries because microbial symbionts attained from the rearing water during the early stages of ontogeny can be maintained into adulthood.

Fusobacteria are commonly identified as a major constituent of freshwater fish microbiomes [22, 78–80]. In our study, Fusobacteria correlated with poor fish condition which was predominately the case for vegetable-fed GIFT. Previous studies have reported that high abundances of Fusobacteria are often associated with carnivorous species [81, 82], likely due to their ability to metabolise protein derived amino acids [83]. Therefore, the possibility that vegetable-fed GIFT are turning to cannibalism could explain the presence of Fusobacteria in fish with poor condition.

## Conclusion

The results from this study contribute to a growing body of work on the influence of diet on the microbiota, trophic status and condition of freshwater fishes. We show that trophic level is not always indicative of a good diet and can represent poor farming practices. Specifically, our results demonstrate how poor feeding practices can negatively impact the success of GIFT farms. We found that fish fed an insufficient vegetable based diet were in a relatively poor condition and while yet to be confirmed, possibly supplementing their diet through filial cannibalism. These results further highlight the extent of challenges faced by low income, small-scale subsistence farmers in developing nations. Small-scale fish farms account for the majority of inland freshwater finfish aquaculture and play a fundamental role in enhancing not only food and income security but also quality of life [2]. For GIFT to contribute to human nutrition and livelihoods in PNG, they need to be farmed productively and profitability. Further research in rural communities in developing nations is needed to improve farming practices through the education of farmers and increased availability of suitable feeds. The introduction of new farming practices such as the use of mono-sex fingerlings stocked by size class could help boost local production of GIFT in PNG. Furthermore, the development of a low-cost feed alternative that better suits the nutritional needs of GIFT would reduce the costs involved with accessing commercial feed pellets, and further increase the farming success and profits of small-scale GIFT farmers in PNG.

## Supporting information

S1 Fig Rarefaction curves. Rarefaction curves of microbial communities sampled from the gastrointestinal tract of Genetically Improved Farmed Tilapia (GIFT) (*Oreochromis niloticus*), a) after sequence quality filtering and without rarefication of sequencing depth, b) equalized sampling depths (34, 313 sequences randomly obtained per sample) and c) after the removal of rare species.
(TIF)

S2 Fig Shannon weaver diversity. Shannon Weaver diversity index of the bacterial communities within the gastrointestinal tract of GIFT fed two different diets. ‘Pellet’ is representative of GIFT fed commercial fish feed pellet based diet, and ‘Vegetable’ is representative of GIFT fed a locally-sourced vegetable-based diet.
(TIF)

S1 Table Sample numbers. Tilapia samples taken in this study for isotopic and microbial analysis, including information about the number of fish taken from each sampling location and the number of samples remaining after quality filtering.
(DOCX)

## References

[1] FAO. The state of world fisheries and aquaculture. (2010) AD, editor. Rome: Food and Agriculture Organization of the United Nations; 2016.

[2] FAO. Meeting the sustainable development goals. Rome: Food and Agriculture Organization of the United Nations; 2018.

[3] Gupta MV, Acosta BO. A review of global tilapia farming practices. Aquaculture Asia. 2004;9:7–12.

[4] Gupta M, Acosta B. From drawing board to dining table: the success story of the GIFT project. NAGA, WorldFish Center Quarterly. 2004;27(3–4):4–14.

[5] Eknath A, Acosta B. Genetic improvement of farmed tilapias (GIFT) project: Final report, March 1988 to December 1997. 1998.

[6] Ansah YB, Frimpong EA, Hallerman EM. Genetically-improved tilapia strains in Africa: Potential benefits and negative impacts. Sustainability. 2014;6(6):3697–721.

[7] Smith PT. Aquaculture in Papua New Guinea: status of freshwater fish farming: Australian Centre for International Agricultural Research (ACIAR); 2007.

[8] Vira H. An analysis of the aquaculture sector in Eastern Highlands Province, Papua New Guinea. [Dissertation]: University of New South Wales; 2015.

[9] Tong S. A sustaliable livelihoods framework-based assessment of the social and economic benefits of fish farming in East New Britain province [Dissertation]: University of New South Wales; 2018.

[10] Smith PT. Small-scale aquaculture in Papua New Guinea: examination of entry points for international aid donors. Enhancing the contribution of small-scale aquaculture to food security, poverty alleviation and socio-economic development. 2013;31.

[11] Narimbi J, Mazumder D, Sammut J. Stable isotope analysis to quantify contributions of supplementary feed in Nile Tilapia *Oreochromis niloticus* (GIFT strain) aquaculture. Aquaculture research. 2018;49(5):1866–74.

[12] De Silva SS, Anderson TA. Fish nutrition in aquaculture: Springer Science & Business Media; 1994.

[13] Cortés E. A critical review of methods of studying fish feeding based on analysis of stomach contents: application to elasmobranch fishes. Canadian Journal of Fisheries and Aquatic Sciences. 1997;54(3):726–38.

[14] Petta JC, Shipley ON, Wintner SP, Cliff G, Dicken ML, Hussey NE. Are you really what you eat? Stomach content analysis and stable isotope ratios do not uniformly estimate dietary niche characteristics in three marine predators. Oecologia. 2020:1–16. 10.1007/s00442-020-04628-6 32179976

[15] Bouillon S, Connolly R, Gillikin D. Use of stable isotopes to understand food webs and ecosystem functioning in estuaries. Treatise on estuarine and coastal science. 2011;7:143–73.

[16] Liu A, Mazumder D, Dove MC, Lai TS, Crawford J, Sammut J. Stable isotope analysis of the contribution of microalgal diets to the growth and survival of pacific oyster *Crassostrea gigas* (Thunberg, 1979) larvae. Journal of Shellfish Research. 2016;35(1):63–9.

[17] Fry B. Stable isotope ecology. New York: Springer; 2006.

[18] Post DM. Using stable isotopes to estimate trophic position: models, methods, and assumptions. Ecology. 2002;83(3):703–18.

[19] Newsome SD, Martinez del Rio C, Bearhop S, Phillips DL. A niche for isotopic ecology. Frontiers in Ecology and the Environment. 2007;5(8):429–36.

[20] Mazumder D, Saintilan N, Wen L, Kobayashi T, Rogers K. Productivity influences trophic structure in a temporally forced aquatic ecosystem. Freshwater Biology. 2017;62(9):1528–38.

[21] Clements KD, Angert ER, Montgomery WL, Choat JH. Intestinal microbiota in fishes: what's known and what's not. Molecular Ecology. 2014;23(8):1891–8. 10.1111/mec.12699 WOS:000333858200001. 24612310

[22] Ghanbari M, Kneifel W, Domig KJ. A new view of the fish gut microbiome: Advances from next-generation sequencing. Aquaculture. 2015;448:464–75. 10.1016/j.aquaculture.2015.06.033 WOS:000360189000059.

[23] Domogala D. Characterization of the Gastrointestinal Tract Microbiome of Tropical Reef Fish. ProQuest Dissertations Publishing: Northern Arizona University; 2016.

[24] Nayak SK. Role of gastrointestinal microbiota in fish. Aquaculture Research. 2010;41(11):1553–73. 10.1111/j.1365-2109.2010.02546.x WOS:000282877000020.

[25] Ganguly S, Prasad A. Microflora in fish digestive tract plays significant role in digestion and metabolism. Reviews in Fish Biology and Fisheries. 2012;22(1):11–6. 10.1007/s11160-011-9214-x

[26] Banerjee G, Ray AK. Bacterial symbiosis in the fish gut and its role in health and metabolism. Symbiosis. 2016;72(1):1–11. 10.1007/s13199-016-0441-8

[27] Adeoye A, Jaramillo-Torres A, Fox S, Merrifield D, Davies S. Supplementation of formulated diets for tilapia (*Oreochromis niloticus*) with selected exogenous enzymes: Overall performance and effects on intestinal histology and microbiota. Animal Feed Science and Technology. 2016;215:133–43.

[28] Hai NV. Research findings from the use of probiotics in tilapia aquaculture: A review. Fish & Shellfish Immunology. 2015;45(2):592–7. 10.1016/j.fsi.2015.05.026. 26003738

[29] Giatsis C, Sipkema D, Smidt H, Heilig H, Benvenuti G, Verreth J, et al The impact of rearing environment on the development of gut microbiota in tilapia larvae. Scientific reports. 2015;5:srep18206. 10.1038/srep18206 PMC4676014 26658351

[30] Fan LM, Chen JZ, Meng SL, Song C, Qiu LP, Hu GD, et al Characterization of microbial communities in intensive GIFT tilapia (*Oreochromis niloticus*) pond systems during the peak period of breeding. Aquaculture Research. 2017;48(2):459–72. 10.1111/are.12894 WOS:000391960400009.

[31] Kinney MJ, Hussey NE, Fisk AT, Tobin AJ, Simpfendorfer CA. Communal or competitive? Stable isotope analysis provides evidence of resource partitioning within a communal shark nursery. Marine Ecology Progress Series. 2011;439:263–76.

[32] Gopi K, Mazumder D, Sammut J, Saintilan N, Crawford J, Gadd P. Isotopic and elemental profiling to trace the geographic origins of farmed and wild-caught Asian seabass (*Lates calcarifer*). Aquaculture. 2019;502:56–62.

[33] Post DM, Layman CA, Arrington DA, Takimoto G, Quattrochi J, Montana CG. Getting to the fat of the matter: models, methods and assumptions for dealing with lipids in stable isotope analyses. Oecologia. 2007;152(1):179–89. 10.1007/s00442-006-0630-x 17225157

[34] Klindworth A, Pruesse E, Schweer T, Peplies J, Quast C, Horn M, et al Evaluation of general 16S ribosomal RNA gene PCR primers for classical and next-generation sequencing-based diversity studies. Nucleic acids research. 2013;41(1):e1 10.1093/nar/gks808 22933715 PMC3592464

[35] Bolger AM, Lohse M, Usadel B. Trimmomatic: a flexible trimmer for Illumina sequence data. Bioinformatics. 2014;30(15):2114–20. 10.1093/bioinformatics/btu170 24695404 PMC4103590

[36] Edgar RC. Search and clustering orders of magnitude faster than BLAST. Bioinformatics. 2010;26(19):2460–1. 10.1093/bioinformatics/btq461 20709691

[37] John OD, Mouatt P, Majzoub ME, Thomas T, Panchal SK, Brown L. Physiological and Metabolic Effects of Yellow Mangosteen (*Garcinia dulcis*) Rind in Rats with Diet-Induced Metabolic Syndrome. International Journal of Molecular Sciences. 2020;21(1):272. 10.3390/ijms21010272 PMC6981489 31906096

[38] Oksanen J, Blanchet FG, Kindt R, Legendre P, Minchin PR, O’Hara R, et al. Package ‘vegan’. Community ecology package, version. 2013;2(9).

[39] R: A Language and Environment for Statistical Computing [Internet]. R Foundation for Statistical Computing. 2017. Available from: https://www.r-project.org/.

[40] Jisr N, Younes G, Sukhn C, El-Dakdouki MH. Length-weight relationships and relative condition factor of fish inhabiting the marine area of the Eastern Mediterranean city, Tripoli-Lebanon. The Egyptian Journal of Aquatic Research. 2018;44(4):299–305.

[41] Le Cren E. The length-weight relationship and seasonal cycle in gonad weight and condition in the perch (*Perca fluviatilis*). The Journal of Animal Ecology. 1951:201–19.

[42] Wickham H. ggplot2: elegant graphics for data analysis: Journal of the Royal Statistical Society: Series A (Statistics in Society); 2016.

[43] Cáceres MD, Legendre P. Associations between species and groups of sites: indices and statistical inference. Ecology. 2009;90(12):3566–74. 10.1890/08-1823.1 20120823

[44] Tichy L, Chytry M. Statistical determination of diagnostic species for site groups of unequal size. Journal of Vegetation Science. 2006;17(6):809–18.

[45] pheatmap: Pretty Heatmaps [Internet]. 2019. Available from: https://cran.r-project.org/package=pheatmap.

[46] Mazumder D, Johansen MP, Fry B, Davis E. Muscle and carapace tissue–diet isotope discrimination factors for the freshwater crayfish *Cherax destructor*. Marine and Freshwater Research. 2018;69(1):56–65.

[47] Rounick JS, Winterbourn MJ. Stable Carbon Isotopes and Carbon Flow in Ecosystems. BioScience. 1986;36(3):171–7. 10.2307/1310304

[48] Trumbore S, Druffel E. Carbon Isotopes for Characterizing Sources and Turnover of Nonliving Organic Matter. The Role of Nonliving Organic Matter in the Earth's Carbon Cycle. 1995;16:7.

[49] Kelly LJ, Martínez del Rio C. The fate of carbon in growing fish: an experimental study of isotopic routing. Physiological and Biochemical Zoology. 2010;83(3):473–80. 10.1086/649628 20201680

[50] Hemre GI, Mommsen TP, Krogdahl Å. Carbohydrates in fish nutrition: effects on growth, glucose metabolism and hepatic enzymes. Aquaculture nutrition. 2002;8(3):175–94.

[51] Maas RM, Verdegem MC, Wiegertjes GF, Schrama JW. Carbohydrate utilisation by tilapia: a meta‐analytical approach. Reviews in Aquaculture. 2020.

[52] Pedrotti FS, Davies S, Merrifield DL, Marques MRF, Fraga APM, Mourino JLP, et al The autochthonous microbiota of the freshwater omnivores jundia (Rhamdia quelen) and tilapia (Oreochromis niloticus) and the effect of dietary carbohydrates. Aquaculture Research. 2015;46(2):472–81. 10.1111/are.12195 WOS:000347377000020.

[53] Mazumder D, Saintilan N, Alderson B, Hollins S. Inputs of anthropogenic nitrogen influence isotopic composition and trophic structure in SE Australian estuaries. Marine pollution bulletin. 2015;100(1):217–23. 10.1016/j.marpolbul.2015.08.047 26371847

[54] Dien LD, Van Sang N, Faggotter SJ, Chen C, Huang J, Teasdale PR, et al Seasonal nutrient cycling in integrated rice-shrimp ponds. Marine Pollution Bulletin. 2019;149:110647.

[55] Vanderklift MA, Ponsard S. Sources of variation in consumer-diet δ 15 N enrichment: a meta-analysis. Oecologia. 2003;136(2):169–82. 10.1007/s00442-003-1270-z 12802678

[56] Beltrán M, FERNÁNDEZ‐BORRÁS J, Médale F, PÉREZ‐SÁNCHEZ J, Kaushik S, Blasco J. Natural abundance of 15N and 13C in fish tissues and the use of stable isotopes as dietary protein tracers in rainbow trout and gilthead sea bream. Aquaculture Nutrition. 2009;15(1):9–18.

[57] McCue MD, Pollock ED. Stable isotopes may provide evidence for starvation in reptiles. Rapid Communications in Mass Spectrometry: An International Journal Devoted to the Rapid Dissemination of Up‐to‐the‐Minute Research in Mass Spectrometry. 2008;22(15):2307–14. 10.1002/rcm.3615 18613003

[58] Council NR. Nutrient requirements of fish and shrimp: National academies press; 2011.

[59] Alam M, Rana Z, Islam S. Comparison of the proximate composition, total carotenoids and total polyphenol content of nine orange-fleshed sweet potato varieties grown in Bangladesh. Foods. 2016;5(3):64. 10.3390/foods5030064 PMC5302402 28231159

[60] Pandi J, Glatz P, Forder R, Ayalew W, Waramboi J, Chousalkar K. The use of sweet potato (*Ipomoea batatas* (L.) Lam) root as feed ingredient for broiler finisher rations in Papua New Guinea. Animal Feed Science and Technology. 2016;214:1–11.

[61] Gannes LZ, O’Brien DM, Del Rio CM. Stable isotopes in animal ecology: assumptions, caveats, and a call for more laboratory experiments. Ecology. 1997;78(4):1271–6.

[62] Macintosh D, De Silva S. The influence of stocking density and food ration on fry survival and growth in Oreochromis mossambicus and O. niloticus female× O. aureus male hybrids reared in a closed circulated system. Aquaculture. 1984;41(4):345–58.

[63] Schwanck E. Filial cannibalism in Tilapia mariae. Journal of Applied Ichthyology. 1986;2(2):65–74.

[64] Fessehaye Y, Kabir A, Bovenhuis H, Komen H. Prediction of cannibalism in juvenile *Oreochromis niloticus* based on predator to prey weight ratio, and effects of age and stocking density. Aquaculture. 2006;255(1–4):314–22.

[65] Fessehaye YRezk M, Bohenvius K, editors. Size dependent cannibalism in juvenile Nile tilapia (*Oreochromis niloticus*)2004.

[66] Chelsky Budarf A, Burfeind D, Loh W, Tibbetts I. Identification of seagrasses in the gut of a marine herbivorous fish using DNA barcoding and visual inspection techniques. Journal of Fish Biology. 2011;79(1):112–21. 10.1111/j.1095-8649.2011.02999.x 21722114

[67] Brown K, DeCoffe D, Molcan E, Gibson DL. Diet-induced dysbiosis of the intestinal microbiota and the effects on immunity and disease. Nutrients. 2012;4:1095–119. 10.3390/nu4081095 23016134 PMC3448089

[68] Tran NT, Xiong F, Hao YT, Zhang J, Wu SG, Wang GT. Two biomass preparation methods provide insights into studying microbial communities of intestinal mucosa in grass carp (*Ctenopharyngodon idellus*). Aquaculture Research. 2017;48(8):4272–83. 10.1111/are.13248 WOS:000404982300025.

[69] Tap J, Furet JP, Bensaada M, Philippe C, Roth H, Rabot S, et al Gut microbiota richness promotes its stability upon increased dietary fibre intake in healthy adults. Environmental Microbiology. 2015;17(12):4954–64. 10.1111/1462-2920.13006 26235304

[70] Sanders JG, Powell S, Kronauer DJ, Vasconcelos HL, Frederickson ME, Pierce NE. Stability and phylogenetic correlation in gut microbiota: lessons from ants and apes. Molecular Ecology. 2014;23(6):1268–83. 10.1111/mec.12611 24304129

[71] Hermans SM, Buckley HL, Case BS, Curran-Cournane F, Taylor M, Lear G. Bacteria as emerging indicators of soil condition. Applied Environmental Microbiology. 2017;83(1):e02826–16. 10.1128/AEM.02826-16 27793827 PMC5165110

[72] Baldo L, Riera JL, Tooming-Klunderud A, Alba MM, Salzburger W. Gut Microbiota Dynamics during Dietary Shift in Eastern African Cichlid Fishes. Plos One. 2015;10(5). 10.1371/journal.pone.0127462 WOS:000354916100145. 25978452 PMC4433246

[73] Elshahed MS, Youssef NH, Luo Q, Najar FZ, Roe BA, Sisk TM, et al Phylogenetic and metabolic diversity of Planctomycetes from anaerobic, sulfide-and sulfur-rich Zodletone Spring, Oklahoma. Applied Environmental Microbiology. 2007;73(15):4707–16. 10.1128/AEM.00591-07 17545322 PMC1951033

[74] Craig S, Helfrich LA, Kuhn D, Schwarz MH. Understanding fish nutrition, feeds, and feeding. 2017.

[75] Brewer TE, Handley KM, Carini P, Gilbert JA, Fierer N. Genome reduction in an abundant and ubiquitous soil bacterium ‘Candidatus Udaeobacter copiosus’. Nature Microbiology. 2017;2(2):1–7. 10.1038/nmicrobiol.2016.198 27798560

[76] Acosta BO, Gupta MV. The Genetic Improvement of Farmed Tilapias Project: Impact and Lessons Learned. In: De Silva SS, Davy FB, editors. Success Stories in Asian Aquaculture Dordrecht: Springer Netherlands; 2010 p. 149–71.

[77] Bledsoe JW, Peterson BC, Swanson KS, Small BC. Ontogenetic Characterization of the Intestinal Microbiota of Channel Catfish through 16S rRNA Gene Sequencing Reveals Insights on Temporal Shifts and the Influence of Environmental Microbes. Plos One. 2016;11(11). 10.1371/journal.pone.0166379 WOS:000387794600054. 27846300 PMC5113000

[78] Roeselers G, Mittge EK, Stephens WZ, Parichy DM, Cavanaugh CM, Guillemin K, et al Evidence for a core gut microbiota in the zebrafish. Isme Journal. 2011;5(10):1595–608. 10.1038/ismej.2011.38 WOS:000295783200005. 21472014 PMC3176511

[79] Larsen A, Mohammed H, Arias C. Characterization of the gut microbiota of three commercially valuable warmwater fish species. Journal of applied microbiology. 2014;116(6):1396–404. 10.1111/jam.12475 24529218

[80] Borsodi AK, Szabo A, Krett G, Felfoldi T, Andras S, Boros G. Gut content microbiota of introduced bigheaded carps (*Hypophthalmichthys spp*.) inhabiting the largest shallow lake in Central Europe. Microbiological Research. 2017;195:40–50. 10.1016/j.micres.2016.11.001 WOS:000392163900006. 28024525

[81] Nelson TM, Rogers TL, Brown MV. The gut bacterial community of mammals from marine and terrestrial habitats. PLoS One. 2013;8(12):e83655 10.1371/journal.pone.0083655 24386245 PMC3875473

[82] Liu H, Guo X, Gooneratne R, Lai R, Zeng C, Zhan F, et al The gut microbiome and degradation enzyme activity of wild freshwater fishes influenced by their trophic levels. Scientific reports. 2016;6 10.1038/srep24340 27072196 PMC4829839

[83] Potrykus J, White RL, Bearne SL. Proteomic investigation of amino acid catabolism in the indigenous gut anaerobe *Fusobacterium varium*. Proteomics. 2008;8(13):2691–703. 10.1002/pmic.200700437 18546150

