# Correction: Diet type influences the gut microbiome and nutrient assimilation of Genetically Improved Farmed Tilapia (*Oreochromis niloticus*)

**DOI:** 10.1371/journal.pone.0251802

**Published:** 2021-05-12

**Authors:** Lara Parata, Debashish Mazumder, Jesmond Sammut, Suhelen Egan

Fig 2 is incorrect. The authors have provided a corrected version here.

**Fig 2:**
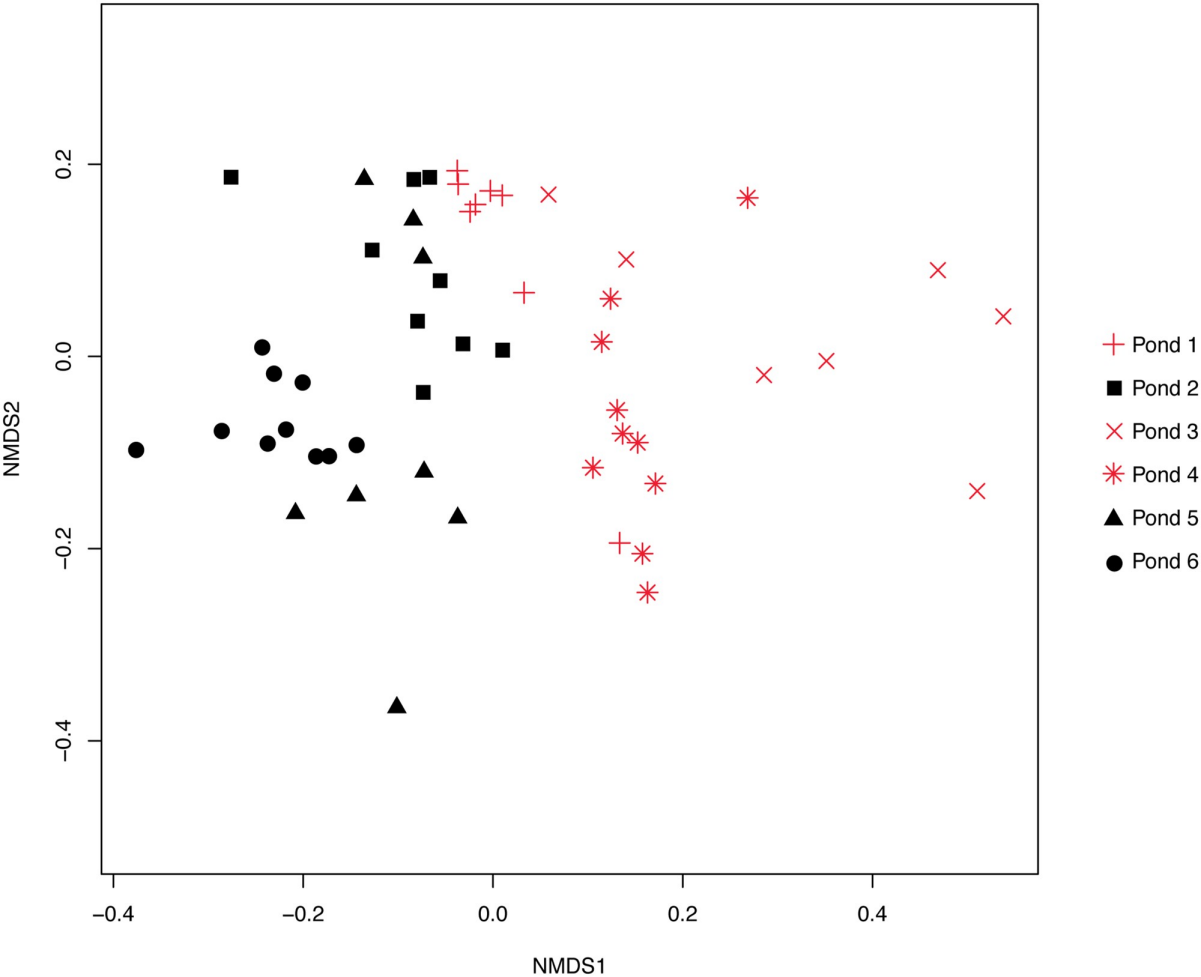
Bray-Curtis dissimilarities of the GIFT microbiome. Non-Metric multidimensional scaling (nMDS) plot based on Bray-Curtis dissimilarity of the bacterial communities of GIFT fed different diets (*P* = 0.001). Pellet-fed fish are represented by solid, black shapes with vegetable-fed fish represented by red symbols. Stress = 0.1703505.

## References

[1] Parata L, Mazumder D, Sammut J, Egan S (2020) Diet type influences the gut microbiome and nutrient assimilation of Genetically Improved Farmed Tilapia (*Oreochromis niloticus*). PLoS ONE 15(8): e0237775. 10.1371/journal.pone.0237775 32813739 PMC7446784

