# Supplementary figures and images for: Diet type influences the gut microbiome and nutrient assimilation of Genetically Improved Farmed Tilapia (Oreochromis niloticus)
Source: PLoS One. 2020 Aug 19;15(8):e0237775. doi: 10.1371/journal.pone.0237775 (PMC7446784; doi:10.1371/journal.pone.0237775)

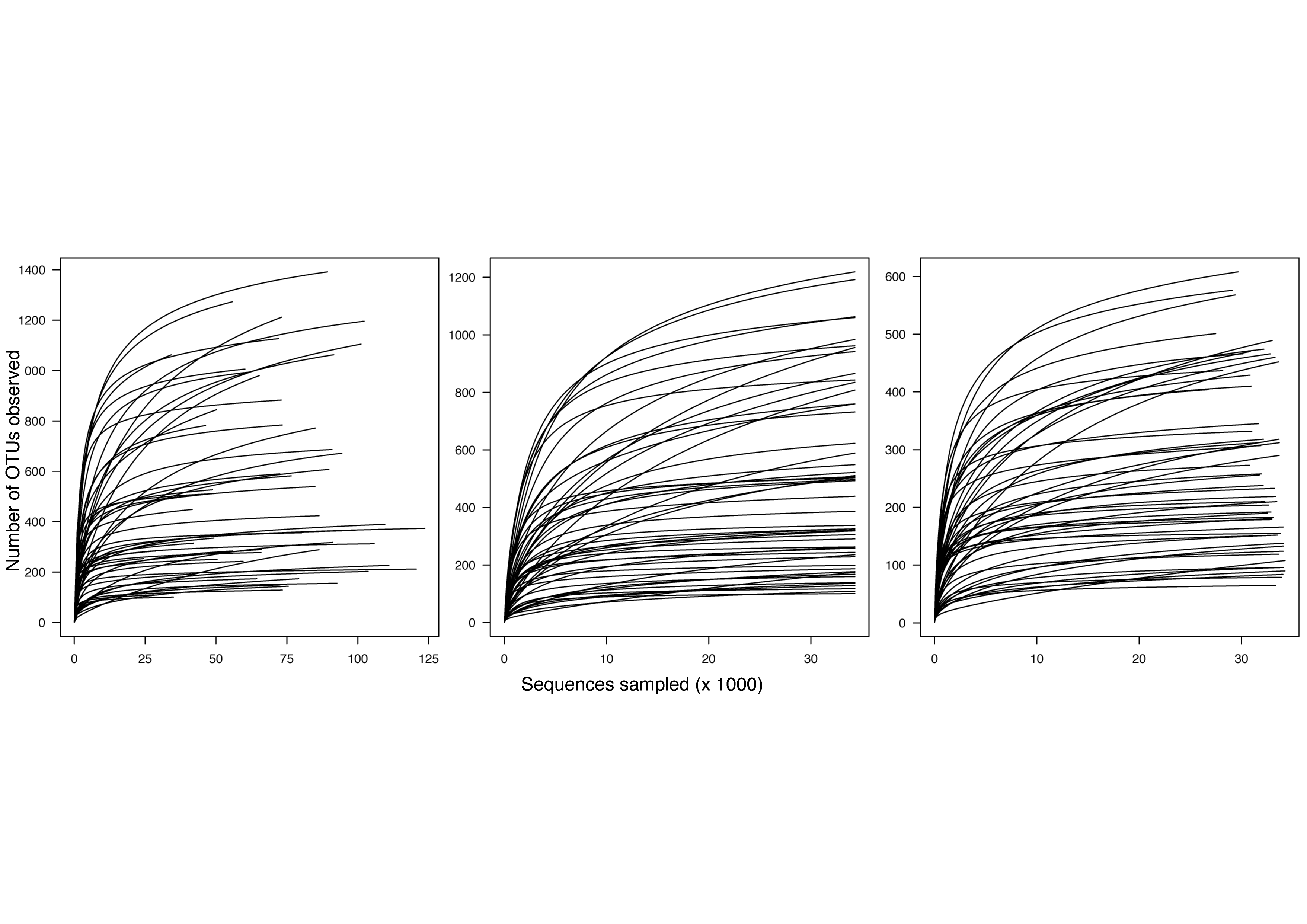

Supplement: S1 Fig — Rarefaction curves of microbial communities sampled from the gastrointestinal tract of Genetically Improved Farmed Tilapia (GIFT) (Oreochromis niloticus), a) after sequence quality filtering and without rarefication of sequencing depth, b) equalized sampling depths (34, 313 sequences randomly obtained per sample) and c) after the removal of rare species. (TIF) [file pone.0237775.s001.tif]

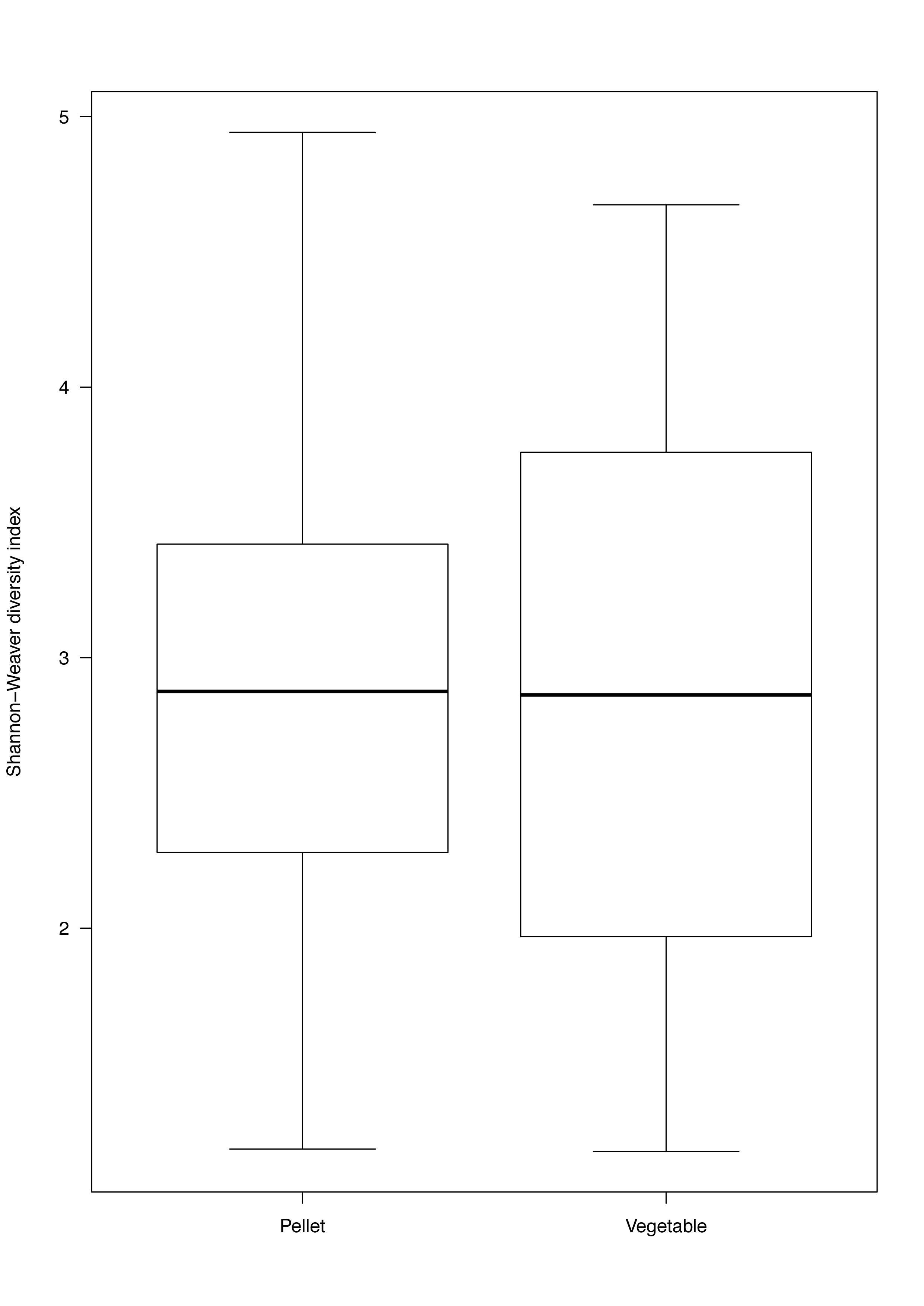

Supplement: S2 Fig — Shannon Weaver diversity index of the bacterial communities within the gastrointestinal tract of GIFT fed two different diets. ‘Pellet’ is representative of GIFT fed commercial fish feed pellet based diet, and ‘Vegetable’ is representative of GIFT fed a locally-sourced vegetable-based diet. (TIF) [file pone.0237775.s002.tif]
