# Supplementary material for: Diet type influences the gut microbiome and nutrient assimilation of Genetically Improved Farmed Tilapia (Oreochromis niloticus)
Source: PLoS One. 2020 Aug 19;15(8):e0237775. doi: 10.1371/journal.pone.0237775 (PMC7446784; doi:10.1371/journal.pone.0237775)
Supplement: S1 Table — Tilapia samples taken in this study for isotopic and microbial analysis, including information about the number of fish taken from each sampling location and the number of samples remaining after quality filtering. (DOCX) [file pone.0237775.s003.docx]

|  | Stable Isotope Analysis | | 16S rRNA gene Sequencing | |
| --- | --- | --- | --- | --- |
|  | Fish sampled (n) | Samples remaining post quality filtering (n) | Fish sampled (n) | Samples remaining post quality filtering (n) |
| Farm 1 | 10 | 10 | 10 | 8 |
| Farm 2 | 10 | 10 | 10 | 9 |
| Farm 3 | 10 | 10 | 10 | 7 |
| Farm 4 | 10 | 10 | 10 | 10 |
| Farm 5 | 10 | 10 | 10 | 8 |
| Farm 6 | 10 | 10 | 10 | 10 |
